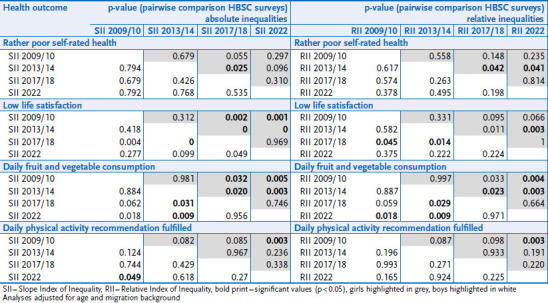# Trends in health inequalities in childhood and adolescence in Germany: Results of the HBSC study 2009/10 – 2022

**DOI:** 10.25646/11876

**Published:** 2024-03-04

**Authors:** Irene Moor, Max Herke, Jenny Markert, Marie Böhm, Franziska Reiß, Ludwig Bilz, Gorden Sudeck, Kristina Winter, for the HBSC Study Group Germany

**Affiliations:** 1 Martin Luther University Halle-Wittenberg, Halle (Saale), Medical Faculty, Interdisciplinary Centre for Health Sciences (PZG), Institute of Medical Sociology; 2 University Medical Centre Hamburg-Eppendorf, Centre for Psychosocial Medicine, Clinic for Child and Adolescent Psychiatry, Psychotherapy and Psychosomatics, Child Public Health Research Section; 3 Brandenburg University of Technology, Cottbus-Senftenberg, Institute of Health; 4 Eberhard Karls University of Tübingen, Institute of Sports Science; 5 Eberhard Karls University of Tübingen, Interfaculty Research Institute for Sport and Physical Activity; 6 Nordhausen University of Applied Sciences, Institute for Social Medicine, Rehabilitation, Sciences and Health Services Research

**Keywords:** SOCIOECONOMIC STATUS, SELF-RATED HEALTH, NUTRITION, PHYSICAL ACTIVITY, LIFE SATISFACTION, HEALTH EQUITY, CHILDREN, ADOLESCENTS, SCHOOLS, HBSC, SURVEY, PREVALENCES, COVID-19, TREND, GERMANY

## Abstract

**Background:**

Many studies have identified health inequalities in childhood and adolescence. However, it is unclear how these have developed in recent years, particularly since the COVID-19 pandemic.

**Methods:**

Analyses are based on the German data from the international Health Behaviour in School-aged Children (HBSC) study from 2009/10 (n = 5,005), 2013/14 (n = 5,961), 2017/18 (n = 4,347), and 2022 (n = 6,475). A total of 21,788 students aged approximately between 11 and 15 years were included. Socioeconomic status (SES) was assessed using the Family Affluence Scale (FAS). Several health indicators were analysed stratified by gender using bivariate and multivariate analysis methods.

**Results:**

In 2022, there are clear socioeconomic inequalities in life satisfaction, self-rated health, fruit and vegetable consumption, and physical activity. These inequalities remained largely constant or increased between 2009/10 and 2022. Between 2017/18 and 2022, no significant changes in inequalities were found.

**Conclusions:**

Health inequalities are persistent and reduce the chances of growing up healthy. There is no evidence that inequalities in the analysed outcomes have changed during the pandemic period (between 2017/18 and 2022). Rather, the changes in the health indicators seem to affect all adolescents in a similar way.

## 1. Introduction

More than one in five children in Germany live in poverty, i.e. they are at risk of income poverty, or their families receive benefits according to the German Social Code (SGB II). This amounts to 2.9 million children and adolescents under the age of 18 [1]. Those affected by poverty often grow up in conditions of considerable deprivation and are restricted in their developmental and educational opportunities as well as in their social participation, for example due to a lack of financial resources for activities or access to experiences. Because of their precarious living conditions, they are more likely to experience shame, marginalisation, and violence than socioeconomically privileged adolescents [1–3]. Particularly in childhood and adolescence, the impact of poverty on educational opportunities or on cognitive [4] as well as social and behavioural development [5] is massive.

In addition, socioeconomic disadvantage in childhood and adolescence is clearly linked to health status, well-being, and health behaviour. These differences in health begin to manifest themselves at an early age [6–9]. Longitudinal studies show that socioeconomic disadvantage in childhood and adolescence also has long-term effects on health in later life [10] and that health inequalities are often perpetuated over the life course.

Adolescents from less privileged socioeconomic backgrounds report significantly more health problems and restrictions, such as poorer mental health or poorer self-rated health, than those from more socially privileged families [11–13]. There are also socioeconomic differences in health and risk behaviours, e.g. students from disadvantaged families often show an unhealthier diet and are less likely to exercise [14, 15]. The extent of socioeconomic differences varies by age, gender, health outcome, and the socioeconomic status (SES) indicator used. Nevertheless, the same pattern often emerges: the lower the SES, the worse the health situation and the less favourable the health behaviour [9, 16, 17]. There are controversial discussions about how to assess socioeconomic status in childhood and adolescence. On the one hand, parental information on their income, education, and occupational status is often used [18]. On the other hand, measures of the adolescents themselves are used, such as on their subjective social status [17, 18], their own education, or the assessment of their family affluence [17, 19, 20].

Regarding the development of health inequalities in childhood and adolescence over time, much research is based on the Health Behaviour in School-aged Children (HBSC) study. International research primarily revealed constant or increasing health inequalities over the last two to three decades [14, 20–22]. For students from Canada, for example, inequalities in life satisfaction and self-rated health increased between 2002 and 2022. Deteriorations in these outcomes were particularly observed among socioeconomically disadvantaged children and adolescents [23]. In the Netherlands, constant socioeconomic differences in mental health were identified from 2001 to 2017 [13]. A study involving up to 37 countries analysed inequalities in psychosomatic health complaints: Between 1994 and 2010, five countries showed increasing, 29 countries showed constant, one country showed decreasing and two countries showed no inequalities [22].

Trends in health inequalities in diet and physical activity between 2002 and 2014 were also examined in a study of 32 countries. In the majority of countries, differences in physical activity and healthy nutrition (daily fruit and vegetable consumption) were observed according to family affluence, to the disadvantage of adolescents with low family wealth. These inequalities mainly remained constant, but in some countries increasing inequalities were observed [14].

For Germany, there are only a few studies analysing health inequalities in children and adolescents over time. Two also refer to HBSC data. These two studies showed that socioeconomic inequalities in both self-rated health and in psychosomatic health complaints remained largely constant between 2002 and 2010 [22, 24]. Further evidence comes from the German Health Interview and Examination Survey for Children and Adolescents (KiGGS). Compared to the baseline survey (2003 – 2006), KiGGS Wave 2 (2014 – 2017) revealed an increase in relative inequalities in self-rated health and soft drink consumption. Although there was an overall positive trend in these health outcomes, this was more pronounced among adolescents with a medium or high social status. In contrast, a reduction in relative inequalities in physical activity was found among boys, as the proportion of less physically active adolescents increased more among medium and high SES boys [8].

It remains unclear how health inequalities among children and adolescents have developed during the COVID-19 pandemic. Some studies suggest that the already deprived and sometimes precarious living conditions of socioeconomically disadvantaged families have worsened significantly and that they have been more affected by the negative effects of the pandemic. Difficult family situations, cramped living conditions during lockdowns, fewer resources for compensation, limited leisure and contact opportunities, fewer resources for home schooling, etc., suggest an increase in health inequalities [25–27]. However, the findings are heterogeneous. For example, an analysis of the Düsseldorf school entry examination between 2018 and 2022 found no increase in inequalities. Instead, negative trends in general health and development were found for all school entrants [28]. Other studies from Lower Saxony found differences in obesity, language development, and recommended deferrals, to the disadvantage of children with a poor educational background. The current prevalences are higher than it would be expected from pre-pandemic data [29]. However, these studies are based on younger children.

Overall, the evidence on the development of health inequalities over time in childhood and adolescence in Germany is heterogeneous and incomplete. The following paper will address this research gap and pursue the following research questions:

1. Are there socioeconomic inequalities in health or health behaviour among adolescents in 2022?
2. Have these health inequalities changed over time from 2009/10 to 2022 (and especially during the pandemic period between 2017/18 and 2022)?
3. Do health inequalities vary by health indicator, age or gender?

**HBSC 2022**

**Data holder:** HBSC Study Group Germany

**Objective:** The aim of the study is to analyse the health and health behaviour of students. Continuous health monitoring through the HBSC study contributes to informing decision-makers in policy and practice about the current fields in prevention and health promotion in childhood and adolescence. A particular focus is on the influencing factors and the social contexts of health in the young generation.

**Study design:** Cross-sectional survey by written questionnaire every four years

**Population:** Students with average ages 11, 13, and 15

**Sampling:** Observation units are schools and the class groups clustered within them. From the population of all state general education schools in Germany, a cluster sample was drawn. In order to obtain a representative estimate (close to the distribution of the population), school size and the percentage distribution of students were included in the sampling, stratified by school type and federal state (Probability Proportional to Size (PPS) design).

**Data collection period:** March – November 2022

**Sample size:**

**2022:** 6,475 students

**All four survey cycles (2009/10 – 2022):** 21,788 students

**HBSC survey cycles:**

**Included in the articles in this issue of the Journal of Health Monitoring:**

▶ 2009/10
▶ 2013/14
▶ 2017/18
▶ 2022

More information can be found at https://hbsc-germany.de/ (German)

## 2. Methods

### 2.1 Sample design and study implementation

The Health Behaviour in School-aged Children (HBSC) study is designed as a cross-sectional survey conducted in schools every four years that covers students aged around 11, 13, and 15 years (mean deviation of 0.5 years). In Germany, these age groups mainly comprise grades 5, 7, and 9. In the school years 2009/10, 2013/14, 2017/18 and in year 2022 the HBSC study surveyed students in general education schools in all 16 federal states of Germany. The schools approached for participation were drawn as a cluster sample from the population of all state general education schools in Germany. In order to obtain a representative estimate (close to the distribution of the population), the school size and the distribution of students were included in the sampling, stratified by school type (Probability Proportional to Size (PPS) design).

The HBSC study is conducted using a questionnaire that the students complete themselves. The study has been approved by the responsible ministries or state education authorities in all federal states (with the exception of North Rhine-Westphalia, where schools decide autonomously whether to participate).

Four survey cycles of the HBSC study Germany were used for the analyses. In addition to the current survey in 2022 (n = 6,475), three further surveys of the following school years were included: 2009/10 (n = 5,005), 2013/14 (n = 5,961), and 2017/18 (n = 4,347). All data sets have been standardised and adjusted by the international HBSC consortium to ensure comparability between the age groups. Further information on the HBSC study and the methodology can be found in the publication by Winter & Moor [30] in this issue of the Journal of Health Monitoring.

**Infobox**

**Slope Index of Inequality (SII) and Relative Index of Inequality (RII)**

**Analysis strategy:** The Slope Index of Inequality (SII) represents absolute inequality, while the Relative Index of Inequality (RII) represents relative inequality. Both measures are based on regression analysis and consider the overall distribution of the socioeconomic measure (in this case the Family Affluence Scale, FAS) as well as the size of the respective socioeconomic groups. The FAS scale was transformed into a metric scale of 0 (highest family affluence) and 1 (lowest family affluence) using Ridit analysis, which was then included as an independent variable in the regression models. The development of absolute and relative health inequalities over time was tested by analysing all survey years together, taking into account an interaction term between SES and the year of data collection [8]. For a more detailed analysis of the changes in inequalities between the respective HBSC surveys, a pairwise comparison (e.g. changes between 2017/18 and 2022) was calculated and repeated using a corresponding interaction term.

**Interpretation:** The absolute inequality (SII) represents a prevalence difference and the relative inequality (RII) a prevalence ratio between students with the lowest and highest family affluence. For example, an SII of 0.20 indicates a prevalence difference of 20 percentage points between those with the lowest and those with the highest family affluence. An SII value of 0.00 indicates no difference in prevalence. An RII of 2.50 can be interpreted as people with the lowest family affluence have a 2.5 times higher risk showing the respective health outcome than those with the highest family affluence. A value of 1.00 would indicate no difference in risk between the groups [8]. Calculation and interpretation of the SII and RII were based on Lampert et al. [8].

### 2.2 Survey instruments

#### Socioeconomic status

There is no standardised instrument for capturing the socioeconomic status of children and adolescents, as their status is still being developed. The international HBSC network has therefore developed an instrument that is easy for students to answer and reflects their family affluence: the Family Affluence Scale (FAS) [31]. This scale has undergone continuous development. The second version (FAS II) for the 2009/10 survey was based on four items (car ownership, own bedroom, vacations taken with the family, computer ownership); for the surveys from 2013/14 onwards, two further items were added to the FAS (FAS III) (number of bathrooms, dishwasher ownership). To ensure comparability, all FAS scales were set to an identical range of values. For the descriptive analyses, they were divided into quintiles, which were grouped into three categories of low (quintile 1 – lower bottom 20 % of the sample), medium (quintiles 2 – 4 – middle 60 % of the sample) and high (quintile 5 – top 20 % of the sample) FAS [32]. For the regression analyses, all FAS scales were transformed into ranks and scaled to a range of values from 0 to 1 for further analyses. This was achieved by dividing each rank value by the maximum rank number. This method allows the data to be interpreted in the context of relative position and is used to determine the Slope Index of Inequality (SII) and the Relative Index of Inequality (RII) (see infobox).

The analysis of health includes indicators of self-rated health and life satisfaction. For health behaviour, dietary habits (fruit and vegetable consumption) and physical activity were evaluated.

#### Life satisfaction

Life satisfaction (LS) is assessed using the ‘Cantril Ladder’ [33]. Based on an eleven-point visual analogue scale (0 – 10) in the form of a ladder, students were asked to rate their life. The bottom of the ladder represents the ‘worst possible life’ (0), the top of the ladder the ‘best possible life’ (10). A low LS was set at 0 – 5 points, a high LS at 6 – 10 points.

#### Self-rated health

The subjective health perception (‘self-rated health’ (SRH)) is assessed using a standardised instrument for recording general well-being and provides information about the future (objective) health of adolescents [34]. Students were asked how they would describe their state of health. The available response categories were ‘excellent’, ‘good’, ‘fair’ and ‘poor’. The first two categories were summarised as rather good SRH and the last two as rather poor SRH.

#### Dietary habits

This article looked at fruit and vegetable consumption as an indicator of healthy dietary habits, as fruit and vegetables consumption has a positive effect on health [35]. The students were asked how often they eat both fruit and vegetables. The response categories ranged from ‘never’, ‘less than once a week’, ‘once a week’, ‘2 – 4 days a week’, ‘5 – 6 days a week’, ‘once a day, every day’ to ‘every day, more than once’. The two indicators were combined into ‘at least daily fruit and vegetable consumption’ (daily consumption must be reported for both fruit and vegetables) and ‘less than daily fruit and vegetable consumption’. The categorisation follows the recommendations of the German Nutrition Society (DGE), which recommends the daily consumption of both fruit and vegetables for a balanced diet [36].

#### Physical activity

Physical activity was operationalised based on the World Health Organization (WHO) recommendation. At the time the study was designed, the recommendation was 60 minutes of daily physical activity for children and adolescents. Although the recommendation has been changed to a weekly average of seven hours since the 2020 update of the WHO recommendations, this operationalisation was chosen as an approximation (see Bucksch et al. [37] in this issue). Children and adolescents were asked how many days of the last seven they had been physically active for at least one hour. It was explained that all physical activities that increase the pulse rate or cause them to be out of breath for some time should be considered and counted together. The response categories ranged from ‘zero’ to ‘7 days’. If at least one hour of physical activity took place on all seven days, this was operationalised as ‘meeting daily physical activity recommendations’. In addition, adolescents who were physically active for less than 60 minutes per day were categorised as ‘not meeting daily physical activity recommendations’.

#### Sociodemographic determinants

Gender and age were considered as sociodemographic determinants. In the 2022 survey, gender was recorded using three response options ‘girl’, ‘boy’ or ‘diverse’. In the previous survey cycles, gender was recorded in binary form (girl, boy). For the trend analyses, students who did not specify their gender or who identified as diverse were excluded from the gender-specific analyses. Age was determined at the time of the survey using the student’s reported month and year of birth and grouped into the age categories ‘11 years’, ‘13 years’ and ‘15 years’ with a deviation of +/- 0.5 years.

### 2.3 Statistical methods

For univariate and bivariate analyses as well as for time trends, prevalences were calculated for the respective health indicators, stratified by survey year, gender, age, and family affluence. Chi-square test was used for bivariate analyses.

The extent of health inequalities based on family affluence was analysed using the SII and the RII (see infobox). As the analysis of trends in health inequalities can differ significantly depending on whether relative or absolute inequalities are analysed, both aspects were considered in the corresponding analyses (analogous to Lampert et al. [8]). The analyses were controlled for age and migration background (information on the measurement instrument can be found in Moor et al. [38]) and the regressions were calculated separately for girls and boys (see infobox for further explanations). Finally, it was tested whether absolute and relative health inequalities had changed significantly over time. First, SII and RII were calculated for this purpose, albeit by pooling the data from all survey years and testing with the help of an interaction term between SES and the year of data collection (p-value is reported). Secondly, a detailed analysis of the change in inequalities between two HBSC surveys (e.g. between 2017/18 and 2022) was carried out, including a corresponding interaction term. In addition, this analysis was repeated with a pairwise pooling of the survey years in order to test for differences in SII and RII between the respective survey years.

A weighting factor was created for all survey cycles to ensure that the sample is nationally representative. This compensates for differences in participation rates across federal states and school types, so that the distribution corresponds to the population. Due to the weighting, all three age categories and the binary gender categories of girls and boys are equally included in the analyses from the 2017/18 survey cycle onwards. For the first time in the 2022 HBSC survey cycle, gender was not recorded exclusively in binary form, with 1.7 % of respondents indicating the category ‘diverse’. In the 2022 data, this distribution was considered in the weighting, while girls and boys were weighted equally (49.2% each; participants who did not specify their gender were excluded). Further details on the weighting of the data can be found in the article by Winter & Moor [30]. Univariate and bivariate analyses were performed using IBM SPSS version 28, multivariate analyses were carried out using the statistical program R [39]. Results with a p-value of less than 0.05 are regarded as statistically significant differences.

## 3. Results

The respective sample distributions by age and gender can be found in the article by Winter & Moor [30], the prevalences for life satisfaction and self-rated health assessment can be found in the article by Reiß & Behn et al. [40] and the frequencies for physical activity in Bucksch et al. [37]. This article focuses on socioeconomic inequalities in these health indicators.

### 3.1 Results on health inequalities in the 2022 survey cycle

**Figure 1:**
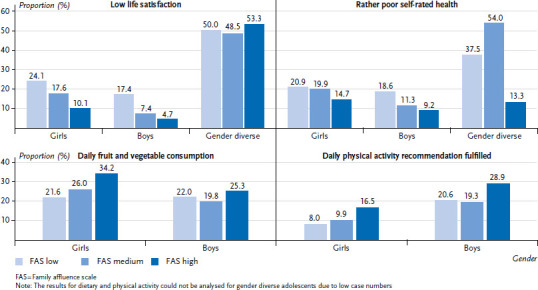
Low life satisfaction, rather poor self-rated health, daily fruit and vegetable consumption, and fulfilment of daily physical activity recommendation (60 min daily) by gender and family affluence (girls n = 2,968 – 3,158, boys n = 2,757 – 2,968, gender diverse = 107 – 108) Source: HBSC Germany 2022

All in all, there is a social gradient in life satisfaction and self-rated health (Figure 1). Girls and boys with low family affluence are significantly more likely to report lower life satisfaction and poorer self-rated health than those with a medium or high family affluence. The difference is considerable: girls with low family affluence are twice as likely, and boys with low family affluence are three times as likely, to report low life satisfaction than their better-off peers. Very high prevalence of low life satisfaction is particularly evident among those belonging to the gender diverse category, regardless of their family affluence (48.5 % – 53.3 %).

With regard to self-rated health, socioeconomic differences are somewhat smaller, but still very clear. Boys with low family affluence were about twice as likely to report rather poor self-rated health, while the difference for girls was six percentage points. Again, the prevalence is significantly higher among gender diverse adolescents, with those with high family affluence showing a lower prevalence comparable to girls and boys.

In terms of dietary habits, the results show that over a third of girls with high levels of family affluence meet the recommendations for daily fruit and vegetable consumption. As family affluence decreases, so does the proportion of girls eating fruit and vegetables daily. Compared to girls, the prevalence of daily fruit and vegetable consumption among boys is lower and the differences by family affluence are small. Only few students meet the WHO’s recommendations for physical activity – especially among girls. There is also a clear social gradient among girls: only half as many girls with a low level of family affluence get enough exercise compared to those with a high level of family affluence. Overall, boys exercise more than girls, but significantly more boys with high family affluence meet the recommendations than those with low or medium family affluence.

**Figure 2:**
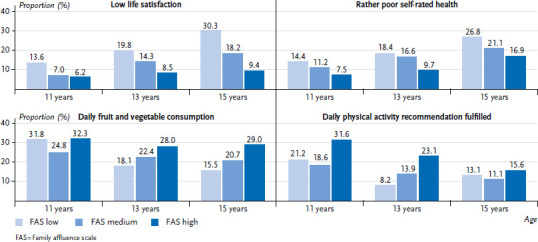
Low life satisfaction, rather poor self-rated health, daily fruit and vegetable consumption, and fulfilment of the daily physical activity recommendation (60 min daily) by age and family affluence (11 years n = 1,903 – 2,037, 13 years n = 1,960 – 2,089, 15 years = 1,933 – 2,072) Source: HBSC Germany 2022

A clear social gradient can also be seen in relation to the age (Figure 2). In each age group, those with low family affluence report lower life satisfaction, poorer self-rated health, less daily fruit and vegetable consumption (except for 11-year-olds), and less daily physical activity. The older the adolescents, the less favourable the outcomes according to family affluence.

### 3.2 Trends in health inequalities (2009/10 – 2022)

**Figure 3:**
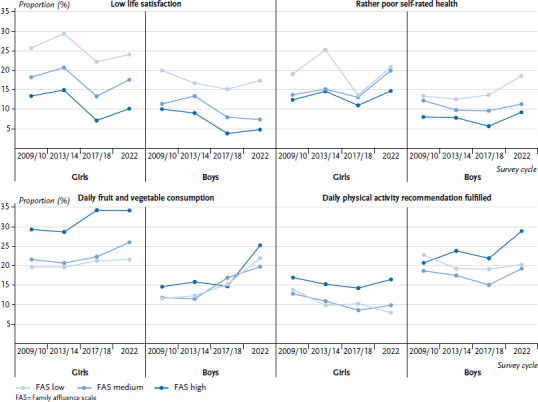
Low life satisfaction, rather poor self-rated health, daily fruit and vegetable consumption, and fulfilment of the daily physical activity recommendation (60 min daily) by gender (girls/boys) and family affluence over time from 2009/10 – 2022 (girls n = 10,466 – 10,677, boys n = 9,982 – 10,136) Source: HBSC Germany 2009/10, 2013/14, 2017/18, 2022

Health inequalities vary by gender and by health outcome (Figure 3). They are particularly evident for life satisfaction in all survey years from 2009/10 to 2022. Even though life satisfaction itself has evolved in various ways in recent years, the extent of inequalities has remained more or less the same. An exception can be seen from 2009/10 to 2013/14 in the form of an improvement in life satisfaction among boys with low family affluence and a simultaneous deterioration in life satisfaction among boys with medium family affluence. This means that health inequalities have decreased over this period. Girl’s life satisfaction follows a similar pattern to that of boys, while the prevalence of low life satisfaction is higher in all survey cycles.

The proportion of adolescents with low self-rated health remained largely stable between 2009/10 and 2017/18. However, a deterioration can be observed in 2022, particularly among girls, but also to a lesser extent among boys. There is also an increase in inequalities for girls between 2009/10 and 2013/14 and again between 2017/18 and 2022. For boys, inequalities increase primarily between 2017/18 and 2022.

There are clear and consistent inequalities in girls’ daily fruit and vegetable consumption. In all survey cycles, girls with a high family affluence are more likely to report daily fruit and vegetable consumption than girls with medium and low family affluence. The gap widened in particular between 2013/14 and 2017/18. For boys, the differences are less pronounced and not noticeable in all survey cycles. However, between 2017/18 and 2022, there was a significant increase in daily fruit and vegetable consumption among boys with the highest family affluence, which occurred to a lesser extent in the other family affluence groups. Accordingly, inequalities in this respect have increased somewhat.

There was also no reduction in inequalities in meeting the physical activity recommendations between 2009/10 and 2022, as these remained largely constant over that period. While overall the prevalence of sufficient physical activity declined similarly for girls in all family affluence groups between 2009/10 and 2017/18, girls became more physically active again in 2022. Except for 2009/10, boys with higher family affluence are more likely to meet the physical activity recommendations than boys with a lower family affluence. The inequalities are most pronounced in 2022. Even more than for girls, boys also show a positive increase in physical activity in 2022.

### 3.3 Extent of relative and absolute health inequalities (2009/10 – 2022)

Absolute (SII) and relative (RII) inequalities were calculated for all health indicators, adjusted for age and migration background, to determine the extent of health inequalities more precisely. They are summarised in Table 1. The results broadly confirm the bivariate results and provide more specific information on the extent. In 2022, there are clear relative inequalities for all the health indicators analysed. Adolescents with low family affluence are more than twice as likely to report rather poor self-rated health (RII_girls_ 2.15; RII_boys_ 2.42), less healthy dietary habits (RII_girls_ 2.46; RII_boys_ 2.39) and less physical activity (RII_girls_ 2.39; RII_boys_ 1.93) than socioeconomically privileged adolescents. In terms of life satisfaction, in 2022 socioeconomically disadvantaged boys have a 6.44 times higher risk of low life satisfaction than socioeconomically more privileged boys (RII 6.44). In 2017/18, the difference was 7.42 times higher. The risk of low life satisfaction was also highest for girls in 2017/18 at 5.81; in 2022, the risk was still 2.69 times higher compared to girls with high family affluence.

The largest absolute inequalities (SII) are found for girls and boys for fruit and vegetable consumption and life satisfaction in 2017/18 and 2022. The difference in prevalence of fruit and vegetable consumption between girls with the lowest family affluence score and those with the highest family affluence score is 22 percentage points (2017/18 and 2022, for boys 18 % and 21 %, respectively). For satisfaction, the difference for girls is 20 (2017/18) and 14 percentage points (2022) and for boys also 14 percentage points (2017/18 and 2022) between those with the highest and lowest family affluence.

Table 2 shows whether the changes between the HBSC survey cycles are significant. It is striking that the greatest significant changes in absolute and relative inequalities occurred primarily between 2013/14 and 2017/18, but not between 2017/18 and 2022 (see Table 2). This means that inequalities in particular increased significantly between 2013/14 and 2017/18 and then remained constant between 2017/18 and 2022. Therefore, it can be seen that for (almost) all adolescents, the prevalences changed for the better between 2017/18 and 2022 in terms of dietary and physical activity, and for the worse in terms of life satisfaction and self-rated health (Figure 3).

## 4. Discussion

### 4.1 Summary of the results

Health inequalities among children and adolescents were analysed using various health indicators for the German 2022 survey cycle. In addition, it was examined how these have changed between 2009/10 and 2022. This included an analysis whether there were gender or age differences in the health indicators considered. Based on the available results of the nationwide HBSC study, it was shown that 1) there are clear inequalities in life satisfaction, self-rated health, fruit and vegetable consumption, and physical activity in 2022; 2) socioeconomic inequalities are evident in all survey cycles with few exceptions and these have largely remained constant or have increased (especially between 2013/14 and 2017/18). Contrary to the assumption, inequalities did not increase between 2017/18 and 2022 (pre- and post-pandemic), but remained at a high level. Rather, the changes seem to apply equally to all status groups; 3) there are differences in the extent of health inequalities by health outcome, gender, and age. The largest relative inequalities were found for life satisfaction (especially for boys) and in absolute terms for fruit and vegetable consumption. No clear social gradient was identified for gender diverse adolescents. However, it became clear that gender diverse adolescents are significantly more likely to have low life satisfaction and poor self-rated health, regardless of their family affluence (except for self-rated health in the case of high family affluence).

### 4.2 Comparison to other research

The findings show that socioeconomic inequalities in health and health behaviour are persistent among children and adolescents. Health inequalities have also been found in a number of other studies [8, 22, 24, 41]. Contrary to our findings, some studies suggest that during the COVID-19 pandemic, socioeconomically disadvantaged adolescents were particularly negatively affected, especially in terms of mental health, including family and school stress [42–45]. However, the results of our study suggest that all children and adolescents were similarly affected by the pandemic, and accordingly both socioeconomically privileged and socioeconomically disadvantaged students reported deterioration in health. This result is consistent with the analysis of the Düsseldorf school entry study by Weyers and Rigó [28], who also found no increase in existing health inequalities in language development and obesity. Rather, unfavourable changes were observed in all children regardless of family affluence.

We also found stable health inequalities in health behaviour, i.e. dietary and physical activity. The existence of health inequalities in dietary and physical activity is not new [8, 14, 15, 46], as dietary and other health-related habits are learned within the family. Studies have shown that families with a low SES are more likely to have unhealthy dietary habits and pass this on to their children [47]. Other studies have also found a correlation between socioeconomic status and physical activity. For example, adolescents with high SES are more likely to be involved in organised sports activities than those with low family affluence [48]. This may be due to the cost of physical activity programmes or other barriers to participation [15]. Interestingly, our results show an improvement in dietary and physical activity between 2017/18 and 2022. There are similar findings available from the MoMo study (motor skills module of the German Health Interview and Examination Survey for Children and Adolescents, KiGGS), which found an increase in physical activity during the pandemic despite the lack of organised sports activities in, e.g., clubs. This may be due to the increase in leisure time during the first lockdown or possibly also due to increased health awareness [49].

Overall, our results show no significant increase in inequalities between 2017/18 and 2022. However, it is clear that differences in health opportunities are strongly linked to socioeconomic background and that socioeconomically disadvantaged children and adolescents have different and, above all, worse starting conditions. This also shows that previous measures to increase health equity are not yet sufficient. Strategies such as the introduction of basic child protection or the establishment of mental health coaches in schools in Germany are important building blocks whose success remains to be evaluated. However, it is clear that these measures need to be both strengthened and sustained in order to achieve the goal of health equity.

Apart from health inequalities, the findings show a significant increase in the prevalence of rather low life satisfaction and rather poor self-rated health compared to 2017/18 (pre-pandemic) and 2022 (post-pandemic). Other studies suggest that the COVID-19 pandemic may have played a central role in this. For example, there is evidence of a link between the events of the pandemic and increased psychological distress, increased depressive symptoms, internalising symptoms, feelings of loneliness, and poorer overall mental health among schoolchildren [50–53]. Overall, the present findings show clear gender differences, with girls in particular reporting higher prevalences of lower life satisfaction and poorer self-rated health than boys. Gender differences in mental health have been observed in many studies [54]. They can be explained in part by the different ways in which girls and boys deal with problems. While boys often tend to use externalising behaviours to deal with problems, girls are more likely to use internalising behaviour, which have implications for mental health [55]. This was also observed in the context of the COVID-19 pandemic, where girls were more likely to respond with a deterioration in their mental health to the negative effects [52, 54]. The evidence on gender diverse adolescents in Germany is rather rudimentary. It is generally assumed that a gender identity outside the binary norm is more likely to lead to fewer opportunities for participation and to discrimination, with negative effects on health [56]. Our findings support this assumption. Even if no conclusions can be drawn regarding the mechanisms of action, it is clear that there is a need for both action and research in this area.

### 4.3 Strengths and limitations of the study

The strengths of the HBSC study are many and are discussed in more detail in the article by Winter & Moor [30]. With regard to the present analysis, the HBSC study made it possible to analyse and compare various health outcomes over a period of twelve years. There are only few studies that allow these analyses over time. To date, there has been little data available to allow analysis of health outcomes for gender diverse children and adolescents.

However, the small number of gender diverse adolescents (n = 112) is a limitation, and the results must be interpreted with caution. Another limitation is that the measurement of socioeconomic status in childhood and adolescence is fraught with uncertainties, as there is no standard indicator. Numerous instruments have been developed to measure both the parental or family situation of adolescents and youth-specific indicators, which play an important role [16, 17]. As part of the HBSC study, the Family Affluence Scale is regularly validated and updated, so it can be assumed to reflect the socioeconomic situation of the family [57, 58]. However, the living conditions and norms of families and adolescents are constantly changing and especially in affluent countries such as Germany, for example, holiday trips or even car ownership may be avoided for environmental reasons rather than economic reasons.

### 4.4 Conclusions

The results of the HBSC study show that not all children and adolescents have the same health opportunities. Their health status still depends on their family background and also varies by gender and age. Overall, it is clear that socioeconomically disadvantaged adolescents, girls and gender-diverse as well as older students need to be particularly the focus of prevention and health promotion strategies. Over the past twelve years, there has been no reduction of health inequalities among adolescents. It appears that the effects of the COVID-19 pandemic are reflected in poorer health outcomes even after restrictions have been lifted. However, crises affect all children and adolescents, and even socioeconomically advantaged families and their children have not been able to fully mitigate the effects. It can be assumed that the effects of the coronavirus crisis will manifest themselves in a variety of ways over the coming years. It is therefore important to monitor health, as the HBSC study does, in order to identify future socioeconomic and health challenges.

## Key statement

- The extent of health inequalities differs depending on the health indicator, gender, and age of the adolescents.
- In 2022, significant inequalities in life satisfaction, self-rated health, fruit and vegetable consumption, and physical activity were identified.
- Between 2009/10 and 2022, health inequalities remained largely constant or have increased, particularly between 2013/14 and 2017/18.
- A comparison of the 2017/18 survey cycle before the COVID-19 pandemic and the 2022 survey cycle shows no increase in inequalities, but they remain at high levels.
- Overall, there was a deterioration in mental health and an improvement in healthy diet and physical activity between 2017/18 and 2022 – these changes affected all adolescents to a similar extent.
- The greatest socioeconomic inequalities are found in the areas of life satisfaction and nutrition.

## Figures and Tables

**Table 1:** Trends of absolute (SII) and relative inequalities (RII) of various health outcomes among girls (n = 10,296 – 10,501) and boys (n = 9,729 – 9,964) Source: HBSC Germany 2009/10, 2013/14, 2017/18, 2022

		2009/10	(95 % CI)	2013/14	(95 % CI)	2017/18	(95 % CI)	2022	(95 % CI)	p trend^#^
**Girls**										
Low	SII	0.12^***^	(0.08 – 0.17)	0.10^***^	(0.05 – 0.15)	0.20^***^	(0.14 – 0.27)	0.14^***^	(0.07 – 0.20)	0.138
life satisfaction	RII	2.33^***^	(1.66 – 3.28)	1.89^***^	(1.34 – 2.67)	5.81^***^	(3.37 – 10.00)	2.69^***^	(1.66 – 4.36)	0.050
Rather poor	SII	0.06^**^	(0.02 – 0.11)	0.07^**^	(0.02 – 0.12)	0.05	(-0.00 – 0.11)	0.11^***^	(0.04 – 0.19)	0.579
self-rated health	RII	1.67^**^	(1.14 – 2.45)	1.73^**^	(1.18 – 2.53)	1.65	(0.97 – 2.81)	2.15^***^	(1.31 – 3.52)	0.985
Daily fruit and	SII	0.1^**^	(0.04 – 0.17)	0.10^**^	(0.03 – 0.16)	0.22^***^	(0.13 – 0.30)	0.22^***^	(0.13 – 0.31)	**0.002**
vegetable consumption	RII	1.51^**^	(1.17 – 1.95)	1.47^**^	(1.13 – 1.91)	2.41^***^	(1.70 – 3.42)	2.46^***^	(1.68 – 3.61)	**0.002**
Daily physical activity	SII	0.04	(-0.01 – 0.08)	0.08^***^	(0.03 – 0.12)	0.05*	(-0.00 – 0.11)	0.08^***^	(0.03 – 0.13)	0.256
recommendation fulfilled	RII	1.37	(0.94 – 2.01)	2.05^***^	(1.36 – 3.09)	1.78*	(0.96 – 3.30)	2.39^***^	(1.33 – 4.27)	0.074
**Boys**										
Low	SII	0.07^**^	(0.03 – 0.12)	0.05^*^	(0.00 – 0.09)	0.14^***^	(0.09 – 0.20)	0.14^***^	(0.07 – 0.20)	**0.008**
life satisfaction	RII	1.91^**^	(1.24 – 2.92)	1.51^*^	(1.02 – 2.22)	7.42^***^	(3.59 – 15.34)	6.44^***^	(2.71 – 15.35)	**0.000**
Rather poor	SII	0.05^*^	(0.01 – 0.09)	0.03	(-0.01 – 0.07)	0.10^***^	(0.05 – 0.16)	0.09^***^	(0.02 – 0.17)	**0.049**
self-rated health	RII	1.61^*^	(1.06 – 2.45)	1.45	(0.95 – 2.21)	3.39^***^	(1.73 – 6.66)	2.42^***^	(1.24 – 4.71)	0.071
Daily fruit and	SII	0.07^*^	(0.01 – 0.13)	0.07^*^	(0.01 – 0.13)	0.18^***^	(0.09 – 0.27)	0.21^***^	(0.12 – 0.29)	**0.001**
vegetable consumption	RII	1.35^*^	(1.02 – 1.78)	1.35^*^	(1.04 – 1.76)	2.19^***^	(1.47 – 3.25)	2.39^***^	(1.64 – 3.48)	**0.001**
Daily physical activity	SII	0.01	(-0.05 – 0.06)	0.07^**^	(0.02 – 0.12)	0.06	(-0.02 – 0.13)	0.11^***^	(0.03 – 0.18)	**0.004**
recommendation fulfilled	RII	1.03	(0.73 – 1.46)	1.54^**^	(1.11 – 2.14)	1.52	(0.88 – 2.62)	1.93^***^	(1.19 – 3.12)	**0.003**

CI = confidence interval, SII = Slope Index of Inequality, RII = Relative Index of Inequality, bold print = significant values

^*^ p ≤ 0.05

^**^ p ≤ 0.01

^***^ p ≤ 0.001

^#^ = significant changes in SII or RII over time

Analyses adjusted for age and migration background

**Table 2:** Information on the significance of changes in absolute (SII) and relative inequalities (RII) over time (pairwise comparison of the HBSC surveys) by gender (girls n = 10,296 – 10,501, boys n = 9,729 – 9,964) Source: HBSC Germany 2009/10, 2013/14, 2017/18, 2022